# Revealing Reaction Mechanisms and Enabling Materials Discovery in Fluxes through Panoramic Synthesis

**DOI:** 10.1063/4.0000911

**Published:** 2025-10-27

**Authors:** Xiuquan Zhou, Hengdi Zhao, Mercouri G Kanatzidis

**Affiliations:** 1 Georgetown University, Washington, DC, USA; 2 Argonne National Laboratory, Lemont, IL, USA; 3 Northwestern University, Evanston, IL, USA

## Abstract

Advancements in many modern technologies, including solar cells, batteries, light-emitting diodes, quantum information science, etc., depend on the continuous discovery of novel materials. Flux, or high-temperature solution, reactions have been widely exploited to synthesize materials such as complex metal oxides, chalcogenides, pnictides, and intermetallics. However, as with most solid-state syntheses, high-temperature flux methods function as a “black box,” offering little insight into solvated species, reaction mechanisms, intermediates, or nucleation processes. In this context, panoramic synthesis^1^ using in situ diffraction techniques is critical for monitoring the dissolution, intermediate phases, and final products in flux reactions. Therefore, through a deeper mechanistic understanding, this approach holds the key to the rational design of synthetic routes leading to new compositional spaces and targeted solid-state materials. We demonstrate that panoramic synthesis, empowered by both synchrotron X-ray and neutron diffraction, can reveal reaction mechanisms in two distinct chemical systems: metal chalcogenides synthesized using molten salts and intermetallics formed in metal fluxes. These mechanistic insights not only facilitate the discovery and design of new materials, even with complex systems featuring unique structures, but also instrumental in developing a novel method for crystal growth. Kanatzidis, M. G., Discovery-Synthesis, Design, and Prediction of Chalcogenide Phases. *Inorganic Chemistry* **2017,** *56* (6), 3158-3173.

